# Logic Analysis of Arrhythmia Triggered by Pacemaker Special Functions - An Educational Presentation

**DOI:** 10.21470/1678-9741-2020-0630

**Published:** 2021

**Authors:** Yi Liu, Xiaojing Yuan

**Affiliations:** 1 Department of Cardiac Diagnosis and Treatment Center, Xuzhou Central Hospital, Xuzhou City, Jiangsu Province, People’s Republic of China.; 2 Department of Cardiac Function, Xuzhou Central Hospital, Xuzhou City, Jiangsu Province, People’s Republic of China.

**Keywords:** Arrhythmias, Cardiac, Pacemaker, Artificial, Electrocardiography, Follow-up Studies

## Abstract

Dual-chamber pacemaker is a fully automatic pacemaker with the function of simulating human physiological pacing. It regulates pacing by programming different refractory periods and various special functions, which are closely related to arrhythmia. After in-depth understanding of these special functions, regular electrocardiogram follow-up analysis is required to provide individualized optimal program control and so is appropriate the administration of the pacemaker’s special functions to better provide optimal clinical guidance for patients with arrhythmia.

**Table:** 

Abbreviations, acronyms & symbols	
**AP**	**= Atrial pacing**
**AR**	**= Atrial refractory**
**ARP**	**=Absolute refractory period**
**AVD**	**= Atrial-ventricular delay**
**CDW**	**= Crosstalk detection window**
**ECG**	**= Electrocardiogram**
**PAVB**	**=Post-atrial ventricular blanking**
**PMT**	**= Pacemaker-mediated tachycardia**
**PVARP**	**= Post-ventricular atrial refractory period**
**PVC**	**= Premature ventricular contraction**
**RRP**	**= Relative refractory period**
**VA**	**= Ventriculoatrial**
**VP**	**= Ventricular pacing**
**VSS**	**= Ventricular safety standby**
**VVP**	**= Ventricular vulnerable period**

## INTRODUCTION

A 38-year-old female patient was implanted with St. Jude dual-chamber pacemaker (Victory XL DR 5816) three years ago, after being diagnosed with viral myocarditis, sinus bradycardia, and atrioventricular block II. After palpitation for one month, the patient came for re-diagnosis and we conducted a pacemaker program control and a 24-hour dynamic electrocardiogram (ECG) tracking.

## QUESTIONS

A What was the cause for palpitation after implantation with the pacemaker?
B What is A pace on the premature ventricular contraction (PVC) option?
C What did the ECG interrogation disclose?

### Discussion of Questions

**Fig. 1:**
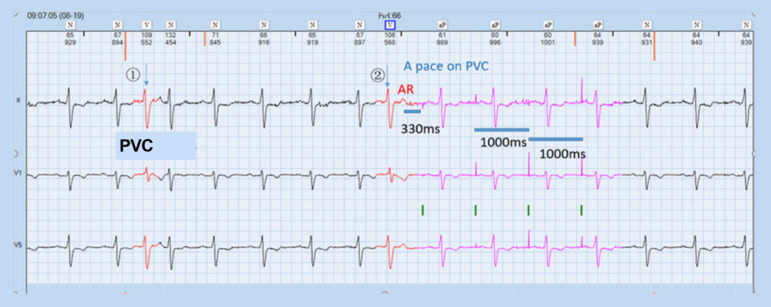
Sinus P wave was transmitted to ventricle and premature ventricular contraction (PVC) occurred. AR=atrial refractory

**Fig. 2:**
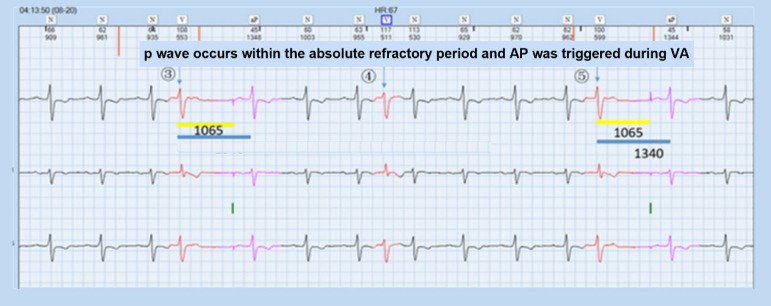
P wave occurred in absolute refractory period and atrial pacing (AP) was released in ventriculoatrial (VA) interval

**Fig. 3:**
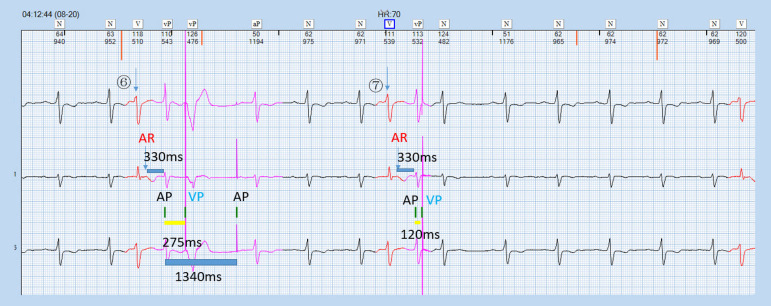
Atrial pacing (AP) overlapped with sinus QRS wave within crosstalk detection window and ventricular safety standby function was triggered. AR=atrial refractory; VP=ventricular pacing

The patient got interposition PVC frequently, triggering A pace on the PVC response of the pacemaker. Resultantly, the sinus P wave lied within the post-atrial ventricular blanking (PAVB), which could not be sensed by the pacemaker, and ventricular pacing (VP) was released in the ventricular vulnerable period (VVP) frequently, contributing to serious arrhythmia. Furthermore, when the sinus P wave occurred in crosstalk detection window (CDW), the ventricular safety standby (VSS) function triggered VP, 120 ms after atrial pacing (AP), aggravating the palpitation (Figures 1 to 3, Question A).

**Fig. 4:**
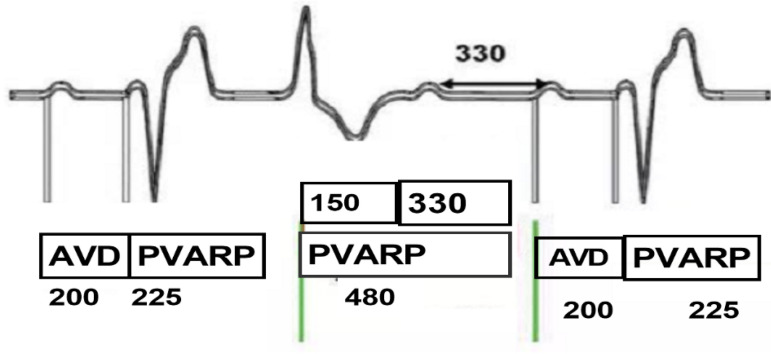
Demonstrations of the special function, A pace on premature ventricular contraction (PVC) of the pacemaker. When PVC occurs, 480-ms post-ventricular atrial refractory period (PVARP) happens thereafter, during which the absolute refractory period is 150 ms and the relative refractory period is 330 ms. If the P wave occurs during the relative refractory period, the P wave is called retrograde P wave and atrial pacing will be released at 330 ms after the retrograde P wave. AVD=atrial-ventricular delay

A pace on PVC option by this specific pacemaker: upon the occurrence of PVC event, the post-ventricular atrial refractory period (PVARP) is extended to 480 ms. The first 150 ms are the absolute refractory period (ARP), which can’t be sensed, and the latter 330 ms are the relative refractory period (RRP). If P wave is sensed within the RRP, named retrograde P wave, AP will be released at 330 ms behind this retrograde P; if P wave is not sensed in the RRP, then AP will be released by the end of ventriculoatrial (VA) interval. Therefore, AP is prevented from being inhibited. Generally, this option was designed to prevent pacemaker-mediated tachycardia (PMT) formed by constant PVC events via extension of PVARP after PVC recognition (Figure 4, Question B).

Through ECG interrogation, pacemaker has aberrant responses to occasional PVC: PVC was marked in Figures 1 and 2, where ① and ④ pointed at it. After PVC, sinus P wave was transmitted to ventricle and PVC recurred at ②. At the end of T wave after this PVC, spontaneous P wave was seen clearly. However, this P wave did not trigger the VP of the pacemaker and it took 720 ms from this PVC to the next AP, shorter than VA interval. At ②, the retrograde P wave occurred within the RRP (330 ms period marked in the Figure 1) after PVC, defined as an atrial refractory (AR) event. Due to A pace on PVC, the pacemaker could sense the retrograde P wave and release AP at 330 ms after AR without triggering VP. ③⑤ displayed a different response from ②. P wave after PVC occurred within the ARP (the former 150 ms in the PVARP interval) and could not be sensed by the pacemaker, which consequently resulted in the AP release during the VA interval. This happened at 04.03 am, at the rest status, with heart beats at 45 times/min, ventriculoventricular interval of 1340 ms, paced atrial-ventricular interval of 275 ms, and VA of 1065 ms. ECG showed the patient had occasional PVC or ventricular premature beat with three typical patterns as below: sinus P wave was transmitted to ventricle, A pace on PVC response by the pacemaker, and AP was triggered during VA interval after PVC. Furthermore, a double pulse appeared after PVC (⑥, Figure 3) and the interval was 275 ms. The first pulse was AP. The QRS wave after PVC occurred within the PAVB interval and remained undetected. Thereafter, VP was released at 275 ms after the AP and occurred in the VVP of sinus QRS. Besides, this AP was 330 ms after the AR event and the P wave was over 280 ms later than the QRS wave. AP-VP sequence was also detected after PVC ⑦ and the interval was 120 ms. The first pulse was AP and was overlapped with the spontaneous sinus QRS wave, which occurred within CDW and thus triggered the VSS function, forming an AP-VS-VP sequence. The pacemaker could not tell whether the signal was crosstalk or spontaneous and released an early VP to avoid asystole. The AR event was 330 ms earlier than the AP and the former QRS wave was over 280 ms earlier than this AR event due to A pace on PVC option. The ECG results disclosed the combined outcomes of both functions of A pace on PVC and VSS of the pacemaker (Question C).

## BRIEF CONSIDERATION OF THE CASE REPORTED

In this case, we finally stopped A pace on PVC option for the patient and also let her take 47.5 mg metoprolol succinate sustained-release tablets once every day for a month and the results proved to be good.

In practice, patients with implantation of earlier pacemakers have chances of PMT, which pushes the advances and enrichments in pacemaker special functions^[1]^. Pacemakers failed to sense during PAVB, thereby inducing the VP release within the VVP, posing high risks of arrhythmia. Producers developed PVARP extension mechanism to avoid detection of the retrograde P wave in order to prevent PMT occurrence^[2]^.

In this case, the option of A pace on PVC was on when the pacemaker identified PVC, extending PVARP, securing the AP to curb the atrial arrhythmia^[3]^. Therefore, A pace on PVC function from St. Jude has been widely used in practice^[4-6]^. Sinus QRS wave appeared within the CDW, the VSS helped to prevent the crosstalk from inhibiting VP. However, clinically, VSS might not only be triggered by the crosstalk sensing, but also frequent PVC events and poor atrial sensing, etc., which encouraged producers to develop automatic ventricular blanking pos-AP, which features dynamic period of program-controlled blanking crosstalk sensing to avoid pseudo-identification of crosstalk. Considering the existence of such complex cases, clinical doctors need to get in-depth understanding of pacemaker mechanisms and value the follow-up programming controls as well as logic analysis in order to enable themselves to customize the optimal programming control for each patient. Only by doing this, clinicians can provide optimal treatment for arrhythmia patients.

## LEARNING POINTS

- PVC response option can help to reduce PVC-triggered PMT by the pacemakers.
- When the PVC response is combined with VSS function, arrhythmia occurs.
- Programming a control specially customized for each patient tends to lower the rate of similar arrhythmia cases.

**Table:** 

Authors' roles & responsibilities	
YL	Substantial contributions to the conception or design of the work; or the acquisition, analysis, or interpretation of data for the work; drafting the work or revising it critically for important intellectual content; final approval of the version to be published
XY	Substantial contributions to the conception or design of the work; or the acquisition, analysis, or interpretation of data for the work; drafting the work or revising it critically for important intellectual content; final approval of the version to be published
